# Fetal cardiac rhabdomyoma due to paternal mosaicism of TSC2: A case report

**DOI:** 10.1097/MD.0000000000021949

**Published:** 2020-08-28

**Authors:** Lin Chen, Yu Jiang, Jing Wang

**Affiliations:** a Department of Obstetrics and Gynecology, West China Second University Hospital; b Key Laboratory of Birth Defects and Related Diseases of Women and Children, Ministry of Education; c Department of Ultrasonography, West China Second University Hospital, Sichuan University, Chengdu, China.

**Keywords:** case report, mosaicism, parallel sequencing, tuberous sclerosis 2, tuberous sclerosis complex

## Abstract

**Rationale:**

Rhabdomyoma is the most common type of fetal heart tumors and 50% to 60% of cardiac rhabdomyomas are associated with tuberous sclerosis complex (TSC). TSC is characterized by hamartomas in multiple organ systems including the brain, heart, skin, lungs, and kidneys, resulting in complications such as learning difficulties, epilepsy, behavioral problems, and renal failure. The etiological diagnosis of Rhabdomyoma is very important.

**Patient concerns:**

A 22-year-old G2P0 woman chose to terminate the pregnancy at 24 + 4 weeks of gestation because of the presence of a cardiac space-occupying lesion in the fetus.

**Diagnoses:**

The pathological diagnosis of cardiac neoplasm tissue was cardiac rhabdomyoma, but the etiology was unknown.

**Interventions:**

Targeted exome capture, next-generation sequencing (NGS) and sanger sequencing were performed on peripheral blood lymphocytes and paternal sperm.

**Outcomes:**

Targeted exome capture sequencing revealed a novel heterozygous variant (NM_000548, c.2294delC) in the tuberous sclerosis 2 (TSC2) gene. Sanger sequencing of maternal blood samples showed no mutation at this locus, however, suspected low level mosaicism was observed in paternal blood samples. Deep NGS analysis showed that about 7% paternal alleles from peripheral blood leucocytes and 20% paternal alleles from sperm carried the mutation consistent with somatic and germinal mosaicism.

**Lessons:**

For fetuses suspected of TSC, when pathogenic mutations are detected in the tuberous sclerosis 1 (TSC1) or TSC2 gene, it is recommended that the parents should be screened by deep NGS and their germ cells are screened as well if necessary, which would help to predict the risk of TSC recurrence in the next pregnancy.

## 1 Introduction

Fetal heart tumors, most of which are benign and rare, account for 2.8% of congenital heart defects. Rhabdomyoma is the most common type of fetal heart tumors, accounting for 60% of cases.^[1]^ Fetal cardiac rhabdomyoma is usually detected 20 weeks post-gestation and frequently occurs in the ventricular cavity. Prognosis depends on tumor size and the presence of other complications, isolated cardiac rhabdomyoma usually indicates better prognosis.^[2–3]^

Cardiac rhabdomyomas are associated with tuberous sclerosis complex (TSC), which account for 50% to 60%of cases. TSC is an autosomal dominant multisystem disorder with a highly variable phenotype characterized by hamartomas in multiple organ systems including the brain, heart, skin, lungs, and kidneys. These changes can result in complications such as learning difficulties, epilepsy, behavioral problems, and renal failure.^[4]^ The incidence of TSC is 1/6000 to 1/1000 in neonates and 1/8000 in adults.^[5]^ TSC is caused by a mutation of the tuberous sclerosis 1 (TSC1) or tuberous sclerosis 2 (TSC2) gene. The 2012 International Tuberous Sclerosis Complex Consensus Recommendations added genetic diagnosis to the diagnostic criteria for TSC, and pointed out that genetic diagnosis can be used as an independent diagnostic criterion.^[6]^ Cardiac rhabdomyoma may be the only manifestation of TSC in fetuses and infants.^[7]^ Therefore, if isolated cardiac rhabdomyoma is found during prenatal examination, clinicians should not only consider the space-occupying effect of the tumor itself and hemodynamic changes, but also conduct further TSC-related gene tests to determine whether the fetus suffers from TSC. In this article, we report a case of fetal cardiac rhabdomyoma due to paternal mosaicism in TSC2.

## 2 Materials and methods

### 2.1 Patient information

**Figure 1:**
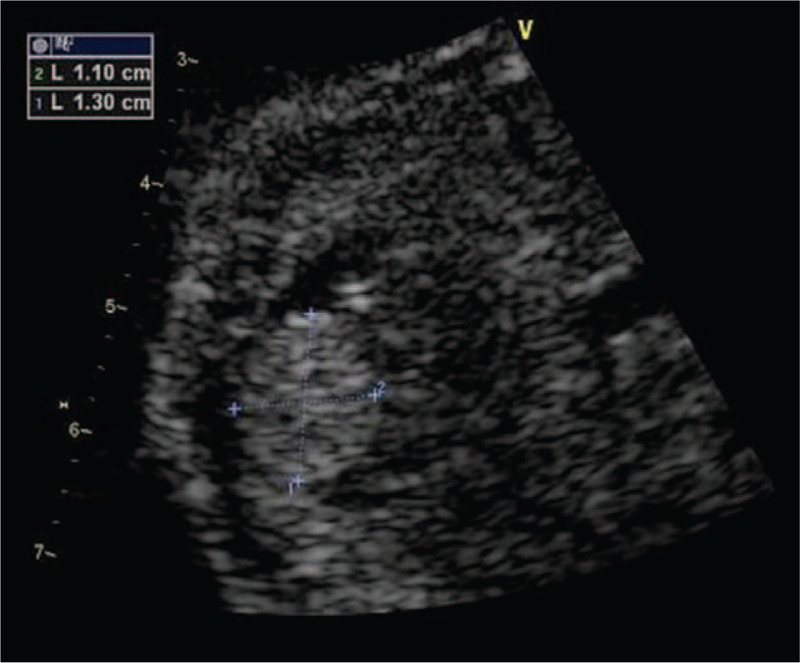
Space-occupying lesion in the fetal heart. The image revealed that a slightly stronger echo (1.1x1.3 cm, no pedicle) was found near the apex of the anterior wall of the right ventricle. The motion of the space occupying lesion was synchronous with ventricular systolic and diastolic.

A 22-year-old G2P0 woman was referred for genetic counseling at 24^+4^ weeks of gestation because of the presence of a cardiac space-occupying lesion in the fetus. This was her second pregnancy, a male fetus. Fetal echocardiographic examination revealed that a slightly stronger echo (1.1x1.3 cm, no pedicle) was found near the apex of the anterior wall of the right ventricle at 24^+4^ weeks of pregnancy. The motion of the space occupying lesion was synchronous with ventricular systolic and diastolic, which was presumed as a probable cardiac rhabdomyoma by ultrasound (Fig. 1). The parents chose to terminate the pregnancy, and then the pathological examination confirmed the diagnosis.

**Figure 2:**
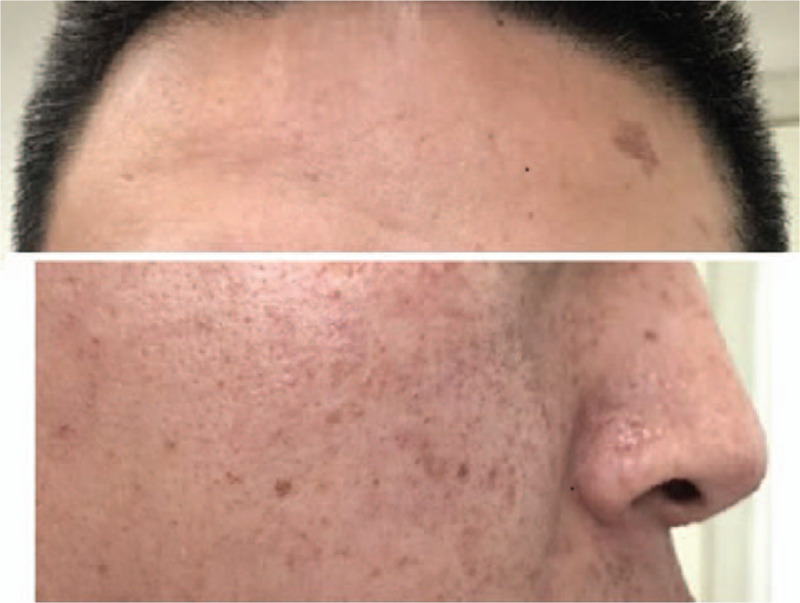
Angiofibroma and café-au-lait spots at face.

**Figure 3:**
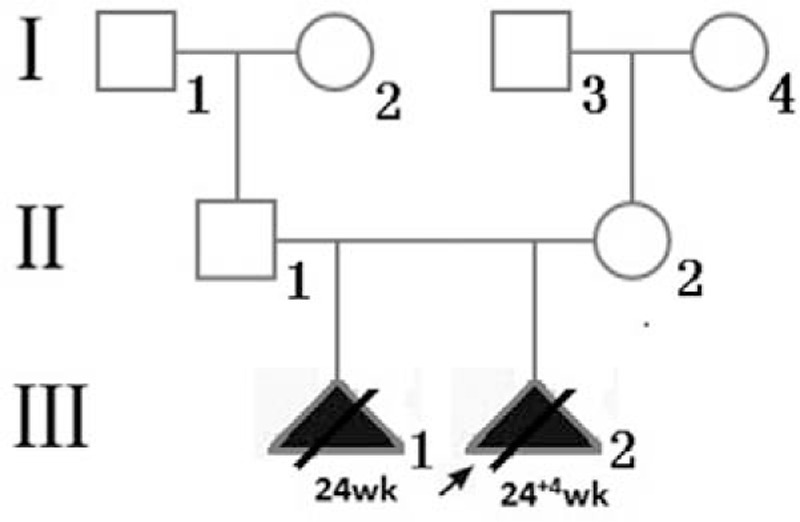
The pedigree of the present family. Open symbols represent healthy individuals. Squares indicate males, circles indicate females, black triangles indicate affected fetuses. The proband is indicated by an arrow.

The woman and her husband were non-consanguineous. The pregnant woman was healthy and her 24-year-old husband showed angiofibroma and café-au-lait spots on his face (Fig. 2), with no abnormalities observed upon examination of the brain, heart, lungs, and kidneys. Two years ago, the couple's first fetus (male) was terminated at 24 weeks of gestation because of multiple space-occupying lesions in the heart and lungs detected through ultrasound. Unfortunately, no genetic and pathological examination was performed on the first fetus and no specimens were preserved. The pedigree of the family is shown in Figure 3.

### 2.2 Laboratory assays and analysis

Genomic DNA was isolated from fetal muscle tissue and the father's sperm using the QIAamp DNA Mini Kit (QIAGEN, Hilden, Germany), and from whole blood using the QIAamp DNA Blood Mini Kit (QIAGEN, Hilden, Germany) following the manufacturer's instructions. For cases III-2 and II-1, targeted exome capture sequencing was performed using the MGP008 targeted exome capture kit (MyGenostics, Beijing, China) (which contained TSC1 and TSC2 genes) according to the manufacturer's protocol. The enrichment libraries were sequenced using a NextSeq 500 sequencer (Illumina, CA). The pathogenic mutation identified by next-generation sequencing (NGS) was confirmed and its origin was investigated by Sanger sequencing of family members’ DNA samples.

## 3 Results

**Figure 4:**
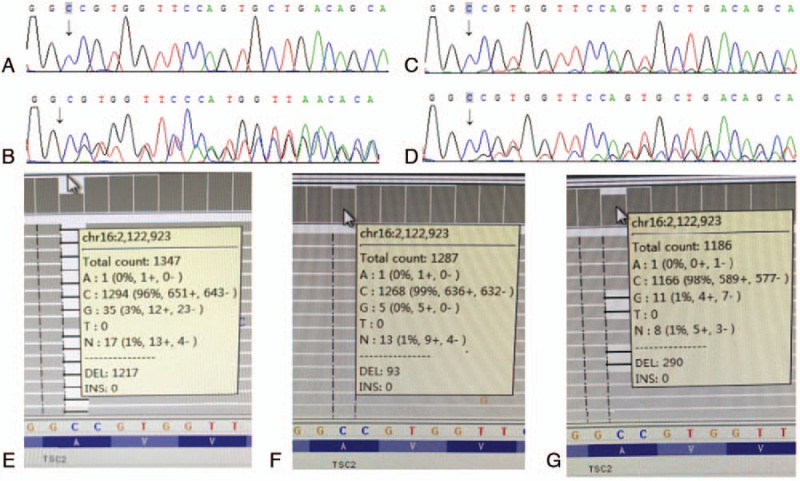
The results of genomic DNA sample sequencing of the family members. Sanger sequencing analysis of the tuberous sclerosis 2 gene revealed no mutationin pregnant woman (A), heterozygous mutation in fetus (B), suspected low level mosaicism in paternal blood samples (C), and mosaicism in paternal sperm samples (D). Deep next-generation sequencing analysis showed that about 49% of the fetal alleles from muscle tissues (E), 7% of the paternal alleles from peripheral blood leucocytes (F), and 20% of the paternal alleles from sperm (G) carried the mutation. Arrows indicate the positions of c.2294. NGS = next-generation sequencing, TSC2 = tuberous sclerosis 2.

Targeted exome capture sequencing revealed a novel heterozygous variant (NM_000548, c.2294delC) in the TSC2 gene of cases III-2, which was confirmed by Sanger sequencing. The c.2294del mutation causes a substitution of the 766^th^ amino acid from valine to tryptophan, and a change in the 770^th^ codon to a stop codon (p.Val766Trpfs∗4). Sanger sequencing of maternal blood samples showed no mutation at this locus; however, suspected low level mosaicism was observed in paternal blood samples. Deep NGS analysis showed that about 7% paternal alleles from peripheral blood leucocytes and 20% paternal alleles from sperm carried the mutation consistent with somatic and germinal mosaicism. The results of genomic DNA sample sequencing of the family members are shown in Figure 4.

## 4 Discussion

TSC is a multisystem disorder with a highly variable phenotype characterized by hamartomas in multiple organ systems including the brain, heart, skin, lungs, and kidneys. TSC also presents variable epidemiology, 2/3 are sporadic, and the remaining 1/3 are familial. The occurrence of TSC is closely related to gene mutation, approximately 10% to 30% cases are due to mutations in the TSC1 gene and the frequency of cases due to mutations of TSC2 gene is higher.^[4]^ Moreover, cases of different epidemiological characteristics may have different genetic mutations. In the sporadic TSC cases, TSC2 gene mutations are nearly as 3 times as TSC1 gene mutations. However, TSC1 gene mutations are more common in the familial cases with autosomal dominant inheritance.^[4–5,8]^ This situation may be caused by that patients with TSC1 have less severe clinical symptoms than patients with TSC2, which increased the chance of forming a family and having children.^[9]^ According to the current literature, almost all mutations of TSC1 gene are nonsense mutations or frameshift mutations, whereas most mutations of the TSC2 gene are missense mutations, deletions, or duplications.^[10]^ In this study, the frameshift mutation of the TSC2 gene produced a truncated protein which caused the fetus to suffer from TSC, yet no study of this variation has been found before.

The TSC patients may have a mosaicism, approximately 15% have somatic mosaicism and about 1% have germline mosaicism.^[11]^ The mosaicism may be 1 of the reasons for the 15% to 20% of TSC patients who have no identifiable mutations.^[4–5]^ Therefore, if TSC-related genes are detected only in peripheral venous blood samples, they may go undetected in mosaic patients, which is the main reason for false negative results of gene testing. Parents with somatic mosaicism usually have mild symptoms, while their affected offspring have more severe clinical symptoms.^[12]^ Parents of germline mosaicism are usually asymptomatic, but 1 or more of their offspring might carry the same mutation.^[13]^ In this study, the proportion of TSC2 mutant cells in sperms is higher than that in peripheral blood from the father. Although the first fetus was not definitely diagnosed as TSC, according to sonographic findings and the father's gene test results, it is speculated that it may also have had TSC.

Detection of mosaicism is an important part of clinical molecular diagnosis of TSC and genetic counseling. The lowest degree of mutant allele was detected by Sanger sequencing range from 15% to 50%. Sanger sequencing alone is not sensitive enough to detect low levels of mosaicism. Parallel sequencing, therefore, remains the only choice. NGS with deep sequence coverage enhances sensitivity and allows accurate quantification of the level of mosaicism.^[14–15]^

For fetuses suspected of TSC, if a pathogenic mutation is detected in the TSC1 or TSC2 gene, it is recommended that their parents be screened for the same gene by deep NGS. If necessary, get their germ cells screened as well. If mosaicism, especially gonadal mosaicism, goes undetected, this might lead to an underestimation of the risk of TSC recurrence in the next pregnancy. Based on the results of genetic testing, one could predict the risk due to family genetics, select appropriate methods for conception, and diagnose the disease prenatally.

## Acknowledgments

We thank our patient and family members who participate in this research program.

## Author contributions

J.W. and L.C. designed the study; J.W., L.C. and Y.J. performed experiments, statistical analyses and interpreted results; J.W. and L.C. wrote the manuscript. All authors reviewed the manuscript.
